# Reliability of an injury scoring system for horses

**DOI:** 10.1186/1751-0147-52-68

**Published:** 2010-12-31

**Authors:** Cecilie M Mejdell, Grete HM Jørgensen, Therese Rehn, Kjersti Fremstad, Linda Keeling, Knut E Bøe

**Affiliations:** 1 Section for Domestic Animal Health and Welfare, National Veterinary Institute, P.O.Box 750 Sentrum, 0106 Oslo, Norway; 2 Department of Animal and Aquacultural Sciences, Norwegian University of Life Sciences, P.O.Box 5003, 1432 Ås, Norway; 3 Department of Animal Environment and Health, Swedish University of Agricultural Sciences, Box 7068, 750 07 Uppsala, Sweden

## Abstract

**Background:**

The risk of injuries is of major concern when keeping horses in groups and there is a need for a system to record external injuries in a standardised and simple way. The objective of this study, therefore, was to develop and validate a system for injury recording in horses and to test its reliability and feasibility under field conditions.

**Methods:**

Injuries were classified into five categories according to severity. The scoring system was tested for intra- and inter-observer agreement as well as agreement with a 'golden standard' (diagnosis established by a veterinarian). The scoring was done by 43 agricultural students who classified 40 photographs presented to them twice in a random order, 10 days apart. Attribute agreement analysis was performed using Kendall's coefficient of concordance (Kendall's *W*), Kendall's correlation coefficient (Kendall's τ) and Fleiss' kappa. The system was also tested on a sample of 100 horses kept in groups where injury location was recorded as well.

**Results:**

Intra-observer agreement showed Kendall's *W *ranging from 0.94 to 0.99 and 86% of observers had kappa values above 0.66 (substantial agreement). Inter-observer agreement had an overall Kendall's *W *of 0.91 and the mean kappa value was 0.59 (moderate). Agreement for all observers versus the 'golden standard' had Kendall's τ of 0.88 and the mean kappa value was 0.66 (substantial). The system was easy to use for trained persons under field conditions. Injuries of the more serious categories were not found in the field trial.

**Conclusion:**

The proposed injury scoring system is easy to learn and use also for people without a veterinary education, it shows high reliability, and it is clinically useful. The injury scoring system could be a valuable tool in future clinical and epidemiological studies.

## Background

Group housing of social animals has obvious advantages for their psychological well-being and in some countries as Sweden, Denmark and Switzerland, legislation requires young horses to be kept in groups. However, in general, group housing of horses is not widely used [1,2]. Perhaps one of the reasons for this is that horse owners are concerned about the risk of injuries caused by kicking and biting, and horses being hurt if chased into obstacles.

The risk of injuries may be substantial when horses are kept in groups [3]. A retrospective study of injured horses treated at the Equine Surgery Clinic at the University of Zürich, Switzerland, showed that the most common cause of injury was a kick from another horse (22%) and that 71% of these injuries occurred on pasture [4]. A Swiss questionnaire answered by horse owners found that 1.7% of the 2912 horses included were treated for injuries and 5.6% of all the diseases and injuries diagnosed by a veterinarian were caused by kicks or bites [5]. In a study on injuries on horses transported for slaughter, 51% of the carcasses had bruises that were ascribed to bites [6]. On the other hand, serious injuries seem to be rare among horses kept in stable social groups [7-9]. It should be noted that injuries may also be caused through play and play fighting, which are considered affiliate social behaviours indicative of positive welfare.

Scoring systems are commonly used in health and welfare assessments in various animal species, such as for lameness, body condition and skin lesions [e.g. [10-14]]. Testing of inter- and intra-observer variation can be used to assure the reliability of the scoring systems [e.g. [15-18]].

Grogan and McDonnell [7] described a system for injury recording in feral horses where categories were ascribed to the horse, not to the injury. In their system, categories were based on a combination of number and severity of injuries and the system can not document the severity of individual injuries. The aim of the present study was to develop and test a tool for recording external injuries on horses kept in groups.

## Methods

### Scoring system

**Figure 1:**
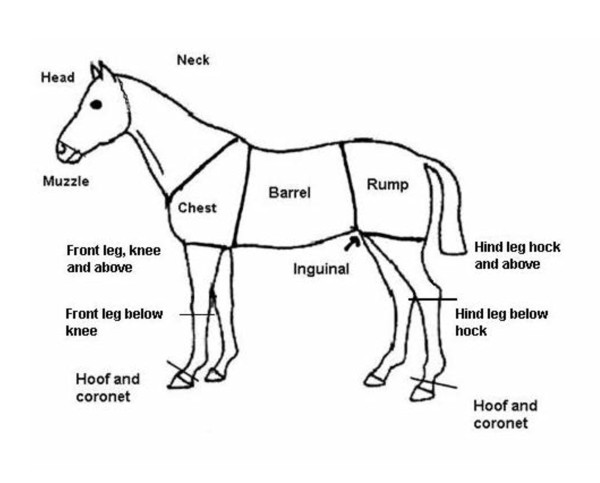
**Sketch of horse used for injury recording, showing names of bodyparts**.

In order to categorize physical injuries in horses, a scoring system was developed based, in part, on earlier work by Grogan and McDonnell [7]. The presence or absence of lacerations and the extent of subcutaneous tissue damage (contusion) were the main factors used to categorize injuries. The draft protocol was piloted under field conditions and was subsequently adjusted to make the categories clearer and easier to interpret. A sketch was used to allocate the injuries to body parts (Figure 1).

**Figure 2:**
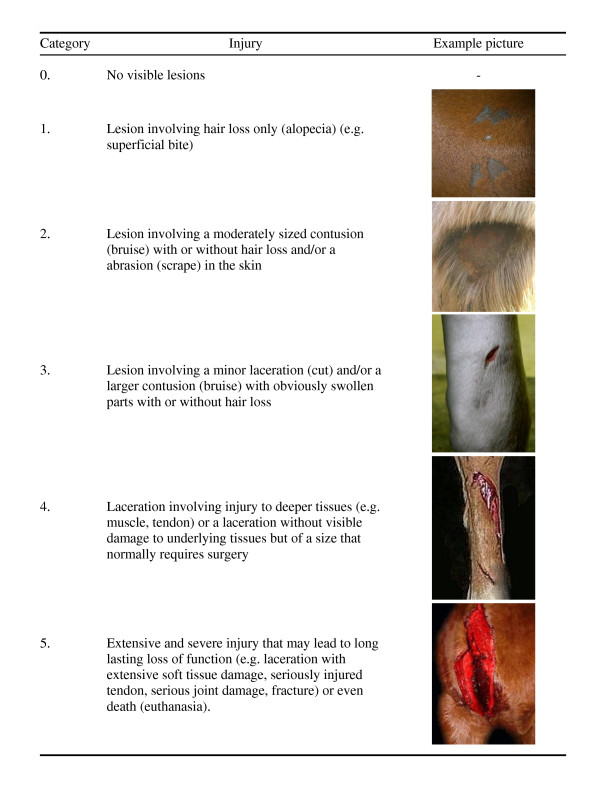
**Descriptions of the different categories within the scoring system**.

The scoring system covered the full spectre from no external injury (category 0), to injury with extensive tissue damage and expected long lasting loss of function (category 5) (Figure 2).

### Observer reliability test

A total of 43 agricultural (i.e. non-veterinary) students from the Swedish University of Agricultural Sciences (N = 26) and the Norwegian University of Life Sciences (N = 17) were recruited to participate in the study. All but two students reported having experience with horses, but only a few had any former experience with injuries. First, the students were assembled for a general introduction, where the scoring system was explained. Examples of lesions (photos) were shown and hung up on a board for later reference. After this introduction the students sat individually at personal computers. They were given written definitions of the categories and a CD with 40 photos of injuries that they were asked to score. Twenty different versions of the CD were made, in which the order of the identical 40 photos was randomised. This was done to compensate for the bias that a preceding photo may have on the score given to the next. The 40 photos covered the categories 1-5 with 5-7 photos from each category and some borderline cases. The 'golden standard' score for each photo was set by a veterinarian (CM), who had defined the categories and selected the photos. In some instances additional information, for example on depth of the wound, was provided as text together with the photo if the information was vital for the judgement of the injury and it was not possible to deduce it from the photo alone. Students were allowed to work for 1.5 h, but most completed within 1 h.

Around ten days later (mean 9.85 days (±1.04 (SE)) the same 43 students each scored the same 40 pictures again, using the same procedure, but now presented in a different order (i.e. another CD). In this way, each picture was scored 86 times.

### Statistical analysis of reliability

Attribute agreement analysis was performed using Kendall's coefficient of concordance (Kendall's *W*), Kendall's correlation coefficient (Kendall's τ) and Fleiss' kappa (Minitab Inc, 2007). Kendall's *W *indicates the degree of association of assessments made by observers when evaluating the same samples. Kendall's τ is used when investigating the agreement between observers and a given standard. *W *and τ values range from 0 (no agreement) to +1 (full agreement). Fleiss' kappa is another statistical measure for assessing the reliability of agreement between observers classifying a number of items. Fleiss' kappa takes into account the degree of agreement that would be expected by chance and kappa values also range from 0 (no agreement) to +1 (full agreement). Since the number of photos within each category was balanced, it was not necessary to use the weighted kappa. Guidelines for interpretation of the strength of kappa values are given by Landis and Koch [19] and are shown in Table 1. Three types of agreement were evaluated; agreement within each observer (intra), agreement between the observers (inter), and agreement between observers and the 'golden standard'. Also, the proportion of the matched scores between all observers and the 'golden standard' was calculated for each injury category. The scorings from the second session (i.e. the observers being more experienced) were used in the analysis of inter-observer agreement and agreement with the 'golden standard'.

**Table 1:** Intra-observer agreement and agreement between observers and the 'golden standard' given as kappa values.

Agreement	Intra-observer % (N)	Each observer versus golden standard % (N)
Slight (0.00-0.20)	0% (0)	0% (0)
Fair (0.21-0.40)	2% (1)	2% (1)
Moderate (0.41-0.60)	12% (5)	30% (13)
Substantial (0.61-0.80)	63% (27)	60% (26)
Almost perfect (0.81-1.00)	23% (10)	7% (3)

The table shows the proportion of observers (in % and number) in different levels of kappa value agreement according to standard description for the strength of agreement [19]

### Field trial

A total of 100 horses were examined for injuries according to the scoring system. The horses were riding horses kept in groups on private premises in Norway. Inclusion criteria were that all horses were kept in groups at least during the day, had been in the group for at least four weeks, and that group size was at least five horses. Five horses from each of 20 groups at 14 different stables were examined for injuries. Composition of the groups varied regarding the number, breed, age, and gender of the horses, as well as to the size of the enclosure and management routines. The study was conducted during the autumn and since the enclosures and paddocks generally did not offer grazing, most horses were fed roughage outdoors. Each horse was led out of the group and thoroughly examined for injuries by a person well trained in the scoring system. Two persons were involved in the injury recording but they did not examine the same horses. Injuries were scored according to the earlier described categories and their location on the body was noted according to Figure 1.

## Results

The Swedish and Norwegian observer groups scoring the photographs of injuries had very similar results; inter-observer Kendall's *W *was 0.92 and 0.90 respectively, and between observers and the golden standard, Kendall's τ was 0.88 and 0.87 respectively. The results from the groups in the two countries are therefore presented together.

### Intra-observer agreement

Kendall's *W *values for intra-observer agreement, that is to say how an individual scored an injury on the first session compared to the second session, were high (range 0.94-0.99). The mean kappa values for the intra-observer agreement was 0.72 (substantial) with a range from 0.40 (fair) to 0.91 (almost perfect) and 86% of the observers had an intra-observer kappa agreement above 0.6 (substantial). The distribution of observers within each level of agreement according to the standard interpretation of strength of kappa-values given by Landis and Koch [19] is shown in Table 1. The proportion of matched scores from first to second session within observers varied from 52.5% to 92.5%.

### Inter-observer agreement

Overall inter-observer agreement (Kendall's *W*) at the second session was 0.91. Kappa values are presented in Table 2. Mean kappa value was 0.59 (moderate). Agreements were substantial for the categories 1 and 5 (0.80 and 0.73, respectively), but lower for the mid categories. The lowest inter-observer agreement was found for category 4.

**Table 2:** Kappa values for inter-observer agreement (left side) and agreement with 'golden standard' (right side) given per injury category and overall

	Agreement between observers			Agreement all observers versus golden standard		
Category	Kappa value	SE	*P *(vs > 0)	Kappa value	SE	*P *(vs > 0)
1	0.80	0.005	<0.0001	0.88	0.024	<0.0001
2	0.54	0.005	<0.0001	0.54	0.024	<0.0001
3	0.49	0.005	<0.0001	0.52	0.024	<0.0001
4	0.38	0.005	<0.0001	0.53	0.024	<0.0001
5	0.73	0.005	<0.0001	0.82	0.024	<0.0001
Overall	0.59	0.003	<0.0001	0.66	0.012	<0.0001

Standard error and significance (kappa different from 0 which refers to what is found by chance) are given.

### Agreement between observers and the 'golden standard'

The percentage of occasions when the observers (second session) gave the same score as the 'golden standard' is presented for each category in Table 3. Agreements were highest for categories 1, 2 and 5. For categories 3 and 4, which had the lowest percentage of matched scores (although still over 50%), observers tended to allocate a lower score (judge the lesions as less serious) than the 'golden standard'.

**Table 3:** Percentage matched scores between observers and the 'golden standard' for each of the injury categories 1-5

		**Scoring by 'golden standard'**				
		**1**	**2**	**3**	**4**	**5**
	
Scoring by observers	1	**91**	8	3	0	0
	2	9	**88**	34	8	0
	3	0	3	**58**	25	2
	4	0	0	5	**54**	13
	5	0	0	1	13	**86**

Kendall's τ values for each observer versus the 'golden standard' varied from 0.79 to 0.95, and the value was 0.88 for all observers versus the 'golden standard'. Kappa values for all observers versus the 'golden standard' per injury category are presented in Table 2. The mean kappa value was 0.66 (substantial). The distribution of kappa values for individual observers versus the 'golden standard' within levels of kappa agreement is shown in Table 1.

### Field study: injuries recorded in horses

Out of a total of 308 injuries recorded in the 100 horses, 78.6% were in injury category 1, 17.5% in category 2, 3.9% in category 3, and 0% in the categories 4 and 5. Twenty-eight horses had no injuries at all, and some horses had many. Young horses and especially young stallions had the highest number of injuries. Details are presented in Table 4.

**Table 4:** Prevalence for injuries in 100 horses from 20 groups.

	Injury category				
	1	2	3	4	5
Total number of injuries	242	54	12	0	0
Number of horses with injury	69	29	9	0	0
Mean number of injuries per horse	3.5	1.8	1.3	0	0
Median number of injuries per horse	1	0	0	0	0
Maximum number of injuries per horse	28	10	3	0	0
Minimum number of injuries per horse	0	0	0	0	0

One horse can have injuries from more than one category

Whereas category 1 injuries were mainly found on the main body including the neck (76.9%), category 2 injuries were commonly found on hind legs (38.9%) and category 3 on the head (25.0%) and legs (58.3%). The barrel and rump of the horse were most exposed to injuries and 49.6% of the category 1 injuries and 43.5% of the total number of injuries where found here. More injuries were scored on hind legs (15.7%) compared to front legs (5.3%).

## Discussion

In the present study, both the intra- and inter-observer agreement of five injury categories scored from photos was generally high, especially for Kendall's coefficients but also for kappa values. Kappa values above 0.40 are considered to be clinically useful [20] and 97% of the observers showed intra-observer agreement as well as agreement with the 'golden standard' above this level. The high agreement with the 'golden standard' suggests the validity of the method and indicates that injury scoring can be standardised in a reliable way, even among persons without veterinary education. After only one hour of training, the reliability of the worst performing observers was fair to moderate and, on average, there was substantial reliability.

When trying to categorize injuries, there will always be borderline cases between two categories, and cases that don't fit all parts of the group description. Thomsen *et al*. [17] addressed this challenge by using the phrase 'in most cases' to make their lameness scoring system for cattle less rigid. This method depends on clinically experienced observers and was not an option in the present study. Instead, we made use of the words 'and' and 'or' in the descriptions, to make them fit a wider range of lesions. The best agreement was found for categories 1, 2 and 5, which can partly be attributed to the fact that categories 1 and 5 have alternatives in only one direction compared to the middle categories. Agreement scores were lowest for category 4. Injuries in this category may be the most difficult ones to evaluate, particularly for non-veterinarians. Severity of an injury depends on the structures and tissues affected and the observers thus need some basic knowledge on equine anatomy and function. If a category 4 or 5 injury is suspected, the normal reaction would be to call a veterinarian in any case.

Category 3 and 4 can be difficult to categorize from photos, especially to determine whether a small lesion is a laceration and the extent of damage to deeper tissues. Erceg *et al*. [21] compared the reliability of a scoring system for clinical cases of chronic discoid lupus erythematosus in humans to scoring of images of the same cases. Even though the correlation between the two was high (0.81), clinical scoring was preferred over images because some important information (e.g. induration) cannot be deduced from a photo. There is reason to suspect that reliability when classifying horse injuries *in situ *might be even better than the current results with photographs, at least among veterinarians and experienced observers.

A number of clinical scoring systems have been tested, and the reliability found in these studies is variable. Kaler *et al*. [14], assessing sheep locomotion from video clips using a 7-point score, found very high average weighted kappa values; 0.93 and 0.91 (almost perfect) for inter- and intra-observer variation, respectively. Other studies show lower agreement. For example, Kristensen *et al*. [16] found that intra-observer and inter-observer weighted kappa values ranged from 0.22 (fair) to 0.75 (substantial) and from 0.17 (slight) to 0.78 (substantial), respectively, for 51 veterinarians scoring body condition twice using a 5-point scale in 20 dairy cattle. In a study by Thomsen and Baadsgaard [13] on lameness and hock and other skin lesions in 283 dairy cows, intra-observer adjusted kappa values varied from 0.55 (moderate) to 0.88 (almost perfect) and inter-observer agreement varied from 0.36 (fair) to 0.84 (almost perfect) for five veterinarians. Burn *et al*. [18] investigated inter-observer reliability among five observers and their trainer, scoring a large number of clinical signs on 40 horses and 40 donkeys, and found low agreement scores for many variables (kappa values and Kendall's *W *below 0.40), especially for donkeys. Only assessment of gender had inter-observer kappa above 0.80 (almost perfect). Thus compared to many other reports, our results show an acceptable high level of observer agreement suggesting that the definitions of the categories were useful. However, our study on selected images avoided the challenge addressed by Burn *et al*. [18] for field studies that a high prevalence of certain findings in a population can result in poor reliability ratings.

The field study with clinical examination of 100 horses kept in groups revealed no serious injuries. In fact 96% of the injuries belonged to category 1 and 2, typically located on the rump and barrel of the horse. The high number of category 1 and 2 injuries found on young horses, suggests that these are caused by play and play fighting and may actually indicate the presence of positive mental states. The most serious injury found on any horse was category 3 (4% of all injuries), which would usually not require veterinary assistance. Interestingly, 25% of the category 3 injuries were found on the head and 48% on the limbs, mainly below carpus/tarsus. A reason for this may be that the skin is tight in these areas and thus more prone to trauma. Forceful kicks on the metatarsus/metacarpus (cannon bone) commonly cause fractures [4].

The lack of serious injuries found in the field study should be interpreted with some caution. Healed injuries (e.g. scars) may be reported as a category 2 if horses, as in this case, were examined only once. The scoring system should therefore primarily be used on acute injuries, i.e. when examining horses on a regular basis.

## Conclusions

The proposed injury scoring shows satisfactory high reliability scores and can be standardised and used in a reliable way. It is relatively easy to learn and use even by people without veterinary clinical experience. However, some basic knowledge on equine anatomy is recommended and training of observers will probably improve reliability scores for categories 3 and 4. We propose that this injury scoring system could be a valuable tool in future clinical and epidemiological studies.

## Competing interests

The authors declare that they have no competing interests.

## Authors' contributions

KB, LK and CM initiated the study. CM drafted the scoring system. LK, TR, GJ and CM designed the observer test. TR, GJ, and CM performed the observer test. KF and GJ recorded field data. TR and LK performed the statistical analysis. CM drafted the manuscript and all authors have read and approved the manuscript.
